# High serum magnesium level is associated with increased mortality in patients with sepsis: an international, multicenter retrospective study

**DOI:** 10.1002/mco2.713

**Published:** 2024-09-17

**Authors:** Le Li, Li Li, Qiuyue Zhao, Xiao Liu, Yaohui Liu, Kailin Guo, Dongsu Zhang, Chang Hu, Bo Hu

**Affiliations:** ^1^ Department of Critical Care Medicine Zhongnan Hospital of Wuhan University Wuhan Hubei China; ^2^ Clinical Research Center of Hubei Critical Care Medicine Wuhan Hubei China

**Keywords:** intensive care unit, mortality, sepsis, serum magnesium

## Abstract

Magnesium imbalances commonly exist in septic patients. However, the association of serum magnesium levels with mortality in septic patients remains uncertain. Herein, we elucidated the association between serum magnesium and all‐cause mortality in septic patients from American and Chinese cohorts by analyzing data from 9099 patients in the Medical Information Mart for Intensive Care‐IV (MIMIC‐IV) database and 1727 patients from a university‐affiliated hospital’ intensive care unit in China. Patients in both cohorts were categorized into five groups based on serum magnesium quintiles from the MIMIC‐IV dataset. Patients with higher serum magnesium levels exhibited an increased risk of 28‐day mortality in both cohorts. The restricted cubic spline (RCS) curves revealed a progressively elevated risk of 28‐day mortality with increasing serum magnesium in MIMIC‐IV cohort, while a J‐shaped correlation was observed in institutional cohort. Our findings have validated the association between high serum magnesium and high mortality in sepsis across different races and medical conditions. Serum magnesium levels might be useful in identifying septic patients at higher mortality risk.

## Graphical abstract

This study investigated the association of serum magnesium with all‐cause mortality in septic patients from American and Chinese cohorts. The findings suggest that high serum magnesium levels at the onset of sepsis are independently associated with increased mortality. Serum magnesium levels might be useful in identifying septic patients at a heightened risk of mortality.

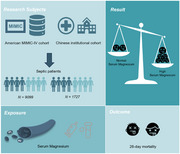

## 1 INTRODUCTION

Sepsis, defined as a life‐threatening organ dysfunction caused by a dysregulated host response to infection, poses a significant global health challenge.^1^ There were about 48.9 million cases reported worldwide in 2017, contributing for 19.7% of total global mortality.^2^ In China, septic patients made up 20.6% of intensive care unit (ICU) admissions, with a staggering 90‐day mortality rate of 35.5%.^3^ Recognizing the gravity of this situation and the enormous and devastating impact of sepsis, the World Health Organization has designated sepsis as a global health priority.^4^, ^5^

Early identification and intervention, including early diagnosis, administration of antimicrobial therapy, hemodynamic support, and fluid resuscitation, are crucial for the treatment of sepsis and septic shock, and these interventions significantly influence patient prognosis.^6^ Additionally, underlying conditions at the time of sepsis diagnosis inevitably affect the prognosis, such as electrolyte imbalance.

Magnesium, ranking as the second most plentiful intracellular cation, is indispensable for enzymatic reactions involved in protein synthesis, energy metabolism, and DNA synthesis.^7^ In clinical and epidemiological studies, serum magnesium could be served as an accessible and cost‐effective biomarker, reflecting magnesium status in humans.^8^, ^9^ Notably, magnesium has been linked to various chronic and inflammatory diseases or conditions.^10^ For instance, hypomagnesemia has been implicated in increasing risks and adverse outcomes among patients with diabetes, cardiovascular disease, depression, and stroke.^11^, ^12^, ^13^, ^14^

Critically ill patients often exhibit hypomagnesemia,^15^ and low levels of serum magnesium were correlated with greater severe conditions and mortality of ICU patients.^16^, ^17^, ^18^, ^19^ Additionally, elevated serum magnesium levels have been correlated to increased mortality in critically ill children, coronavirus disease 2019 (COVID‐19) patients, and hospitalized individuals.^20^, ^21^ However, there is a dearth of studies investigating the relationship between serum magnesium levels at the onset of sepsis and mortality in patients. Furthermore, it remains unknown whether this association exhibits heterogeneous due to different ethnicities.

Therefore, our study aimed to assess the relationship between serum magnesium levels at the onset of sepsis and mortality rates utilizing data from both the American Medical Information Mart in Intensive Care‐IV (MIMIC‐IV) cohort and the Chinese institutional cohort.

## 2 RESULTS

**FIGURE 1:**
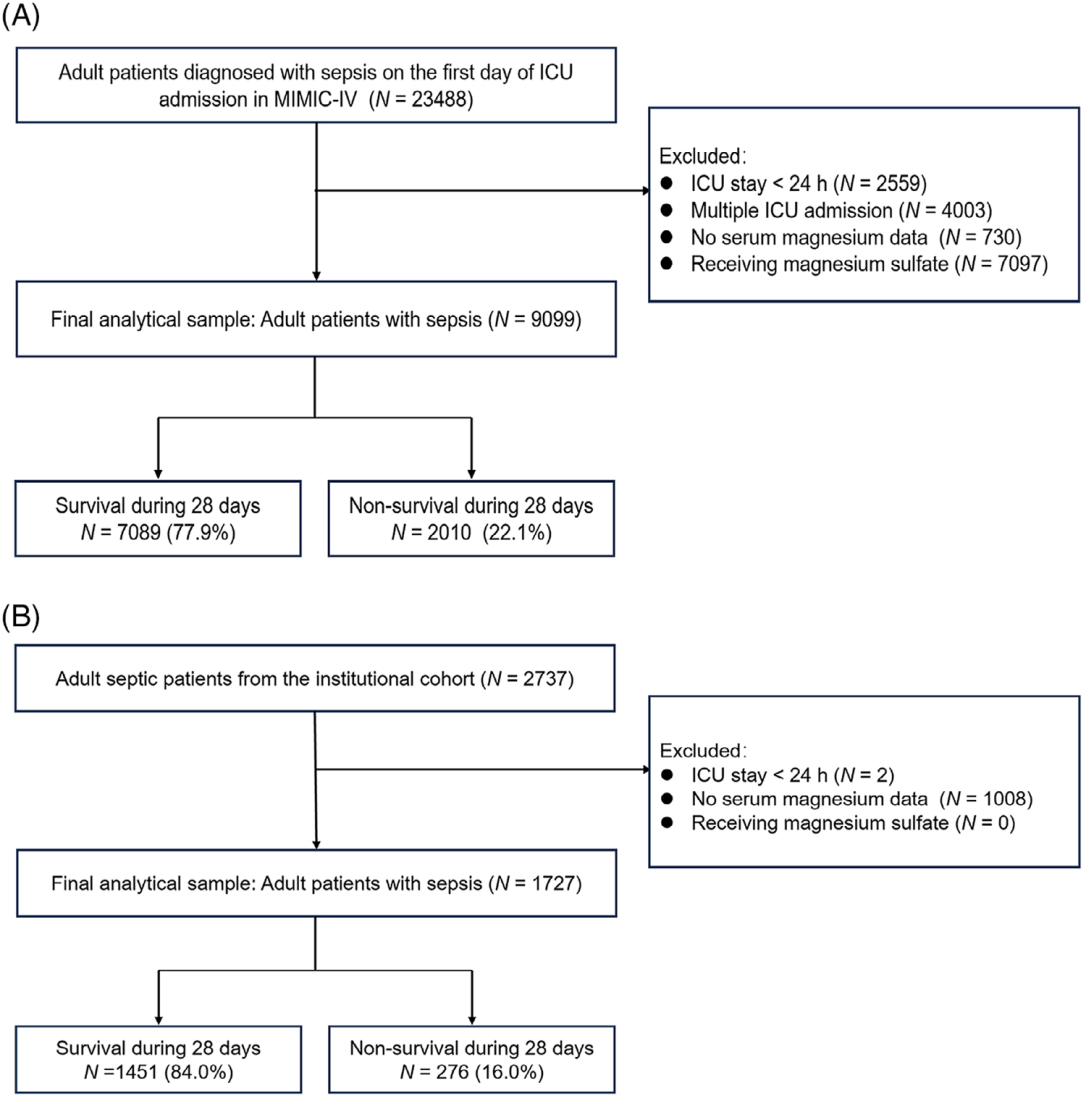
(A, B) Detailed process of data extraction. Detailed process of data extraction of the Medical Information Mart for Intensive Care‐IV (MIMIC‐IV) cohort (A) and the institutional cohort (B). ICU, intensive care unit.

A total of 9099 patients were enrolled from American MIMIC‐IV cohort, while the Chinese institutional cohort comprised 1727 patients (Figure 1). Patients in both cohorts were divided into five groups, categorized by the serum magnesium quintiles from the MIMIC‐IV dataset (MIMIC‐IV cohort: Q1–Q5; institutional cohort: G1–G5).

### 2.1 Baseline characteristics of participants

The median serum magnesium level was 0.86 mmol/L [interquartile range (IQR): 0.82–0.99] (Table 1) in the MIMIC‐IV cohort and 0.87 mmol/L (IQR: 0.76–1.01; Table S1) in the institutional cohort. Participants with higher magnesium levels (Q4–Q5, G4–G5 groups) in both cohorts exhibited higher disease severity scores and worse laboratory parameters. However, patients with sepsis in the MIMIC‐IV cohort exhibited a higher prevalence of complications, while the institutional cohort demonstrated a more severe illness profile, as evidenced by higher sequential organ failure assessment (SOFA) score and worse vital signs (Table S2).

**TABLE 1:** Characteristics and outcomes of participants categorized by serum magnesium in the American Medical Information Mart for Intensive Care‐IV (MIMIC‐IV) cohort.

	Total	Serum total magnesium (mmol/L)					
Characteristics	(0.37–8.64)	Q1 (<0.78)	Q2 (0.78–0.85)	Q3 (0.86–0.90)	Q4 (0.91–1.03)	Q5 (>1.03)	*p* value
Number of patients, *n*	9099	1170	2137	2418	1966	1408	
**Baseline variables**							
Age (year)	69.3 (57.9–80.0)	69.1 (57.2–80.6)	68.9 (56.7–80.5)	69.1 (57.5–80.0)	69.1 (58.4–79.6)	70.4 (59.5–79.9)	0.393
Female	3675 (40.4)	536 (45.8)	935 (43.8)	923 (38.2)	738 (37.5)	543 (38.6)	<0.001
Ethnicity, *n* (%)							0.071
White	6040 (66.4)	779 (66.6)	1433 (67.1)	1613 (66.7)	1306 (66.4)	909 (64.6)	
Asian	278 (3.1)	28 (2.4)	52 (2.4)	75 (3.1)	74 (3.8)	49 (3.5)	
Black	864 (9.5)	123 (10.5)	199 (9.3)	220 (9.1)	186 (9.5)	136 (9.7)	
Hispanic or Latino	277 (3.0)	44 (3.8)	78 (3.6)	72 (3.0)	40 (2.0)	43 (3.1)	
Other/unknown	1640 (18.0)	196 (16.8)	375 (17.5)	438 (18.1)	360 (18.3)	271 (19.2)	
**Vital signs**							
Heart rate (b/min)	85 (75–96)	86 (76–99)	85 (74–97)	85 (75–97)	84 (75–95)	83 (75–94)	<0.001
SBP (mmHg)	114 (105–125)	115 (106–127)	115 (106–128)	114 (106–126)	113 (105–123)	112 (104–121)	<0.001
DBP (mmHg)	60 (54–67)	61 (55–69)	62 (55–68)	61 (55–68)	60 (54–67)	58 (52–64)	<0.001
MAP (mmHg)	57 (50–64)	57 (50–65)	58 (51–65)	58 (51–64)	57 (50–63)	56 (49–62)	<0.001
Respiratory rate (b/min)	19 (17–22)	19 (17–22)	19 (17–22)	19 (17–22)	19 (17–23)	19 (17–22)	0.096
Temperature (°C)	36.8 (36.6–37.2)	36.9 (36.7–37.3)	36.9 (36.7–37.2)	36.9 (36.6–37.2)	36.8 (36.5–37.1)	36.7 (36.4–37.0)	<0.001
**Laboratory parameters**							<0.001
Cr (mg/dL)	1.3 (0.9–2.3)	1.1 (0.8–1.7)	1.1 (0.8–1.8)	1.2 (0.9–2.1)	1.5 (1.0–2.5)	1.9 (1.2–3.2)	<0.001
BUN (mg/dL)	28 (18–49)	22 (15–33)	25 (17–39)	28 (17–47)	33 (20–58)	44 (23–77)	<0.001
WBC (K/L)	13.7 (9.8–18.7)	12.9 (9.0–17.6)	12.9 (9.1–18.0)	13.4 (9.8–18.3)	14.1 (10.4–19.4)	15.2 (10.9–20.2)	<0.001
Hemoglobin (g/L)	9.7 (8.2–11.3)	9.9 (8.4–11.5)	10.0 (8.4–11.6)	9.9 (8.4–11.4)	9.5 (8.2–11.2)	9.0 (7.8–10.6)	<0.001
Platelets (K/µL)	168 (114–237)	177 (119–248)	176 (123–245)	174 (120–238)	166 (112–230)	146 (100–219)	<0.001
PT (s)	14.8 (13.0–18.3)	14.5 (12.8–17.8)	14.4 (12.7–17.4)	14.5 (12.9–17.7)	15.1 (13.2–18.7)	16.0 (13.7–21.3)	<0.001
PTT (s)	32.7 (28.3–43.1)	31.7 (27.6–40.7)	31.7 (27.6–39.7)	32.4 (28.3–42.3)	33.2 (28.4–45.4)	35.7 (30.2–52.9)	<0.001
INR	1.3 (1.2–1.7)	1.3 (1.1–1.6)	1.3 (1.1–1.6)	1.3 (1.2–1.6)	1.4 (1.2–1.7)	1.5 (1.2–2.0)	<0.001
Potassium (mEq/L)	4.5 (4.2–5.1)	4.4 (4.0–4.9)	4.4 (4.1–4.9)	4.5 (4.2–5.0)	4.6 (4.3–5.2)	4.8 (4.4–5.5)	<0.001
Chloride (mmol/L)	106 (102–110)	106 (102–109)	106 (102–110)	106 (101–110)	106 (101–110)	107 (102–111)	<0.001
Sodium (mEq/L)	140 (137–143)	140 (137–142)	140 (137–143)	140 (137–143)	140 (137–143)	140 (137–143)	<0.001
Calcium (mEq/L)	8.6 (8.1–9.0)	8.4 (7.8–8.9)	8.5 (8.1–9.0)	8.6 (8.1–9.0)	8.6 (8.1–9.1)	8.6 (8.2–9.2)	<0.001
Phosphate (mEq/L)	3.9 (3.2–4.9)	3.5 (2.9–4.2)	3.7 (3.1–4.5)	3.9 (3.2–4.8)	4.1 (3.4–5.2)	4.5 (3.5–6.1)	<0.001
Glucose (mg/dL)	166 (131–217)	153 (120–208)	156 (124–204)	163 (128–211)	173 (139–225)	182 (149–236)	<0.001
**Score system**							<0.001
SOFA	3 (2–4)	3 (2–4)	3 (2–4)	3 (2–4)	3 (2–5)	4 (3–6)	<0.001
SAPSII	40 (32–49)	36 (29–45)	37 (30–47)	39 (31–49)	41 (34–51)	45 (37–56)	<0.001
APSIII	49 (37–64)	44 (35–57)	46 (35–59)	48 (36–63)	51 (39–66)	56 (42–75)	<0.001
OASIS	34 (28–40)	33 (27–39)	33 (28–39)	34 (29–40)	35 (29–40)	35 (30–42)	<0.001
GCS	14 (9–15)	14 (10–15)	14 (9–15)	14 (9–15)	13 (9–15)	14 (8–15)	0.094
**Interventions, *n* (%)**							
Ventilation	7142 (78.5)	844 (72.1)	1633 (76.4)	1907 (78.9)	1592 (81.0)	1166 (82.8)	<0.001
RRT	671 (7.4)	76 (6.5)	110 (5.1)	164 (6.8)	157 (8.0)	164 (11.6)	<0.001
Vasopressor	3726 (40.9)	350 (29.9)	726 (34.0)	922 (38.1)	916 (46.6)	812 (57.7)	<0.001
**Comorbidities, *n* (%)**							
COPD	2640 (29.0)	310 (26.5)	586 (27.4)	726 (30.0)	617 (31.4)	401 (28.5)	0.011
Hypertension	6017 (66.1)	743 (63.5)	1345 (62.9)	1586 (65.6)	1348 (68.6)	995 (70.7)	<0.001
Congestive heart failure	3216 (35.3)	330 (28.2)	630 (29.5)	860 (35.6)	812 (41.3)	584 (41.5)	<0.001
Liver disease	1656 (18.2)	189 (16.2)	358 (16.8)	394 (16.3)	377 (19.2)	338 (24.0)	<0.001
Renal failure	2574 (28.3)	286 (24.4)	510 (23.9)	643 (26.6)	616 (31.3)	519 (36.9)	<0.001
Cerebrovascular disease	1383 (15.2)	217 (18.5)	356 (16.7)	344 (14.2)	270 (13.7)	196 (13.9)	<0.001
Diabetes	2997 (32.9)	404 (34.5)	649 (30.4)	748 (30.9)	688 (35.0)	508 (36.1)	<0.001
Malignant cancer	1282 (14.1)	181 (15.5)	338 (15.8)	330 (13.6)	269 (13.7)	164 (11.6)	0.005
**Primary outcome**							
28‐day mortality, *n* (%)	2010 (22.1)	208 (17.8)	427 (20.0)	507 (21.0)	477 (24.3)	391 (27.8)	<0.001
**Secondary outcome**							
90‐day mortality, *n* (%)	2658 (29.2)	303 (25.9)	589 (27.6)	666 (27.5)	611 (31.1)	489 (34.7%)	<0.001
ICU mortality, *n* (%)	1124 (12.4)	95 (8.1)	213 (10.0)	279 (11.5)	291 (14.8)	246 (17.5)	<0.001
In‐hospital mortality, *n* (%)	1612 (17.7)	154 (13.2)	334 (15.6)	403 (16.7)	388 (19.7)	333 (23.7)	<0.001
ICU length of stay, day	3.0 (1.8–5.8)	2.9 (1.8–5.2)	2.9 (1.8–5.5)	2.9 (1.8–5.7)	3.2 (1.9–6.1)	3.4 (1.9–6.1)	<0.001
Hospital length of stay, day	8.4 (5.1–14.7)	7.8 (4.9–13.2)	8.0 (4.9–14.3)	8.5 (5.1–14.6)	8.6 (5.2–14.9)	9.2 (5.3–16.1)	<0.001

**Abbreviations**: APSIII, Acute Physiology and Chronic Health Evaluation III; BUN, blood urea nitrogen; COPD, chronic obstructive pulmonary disease; Cr, creatinine; DBP, diastolic blood pressure; GCS, Glasgow Coma Scale; ICU, intensive care unit; INR, international normalized ratio; MAP, mean arterial pressure; OASIS, Oxford Acute Severity of Illness Score; PT, prothrombin time; PTT, partial thromboplastin time; RRT, renal replacement therapy; SAPSII, Simplified Acute Physiology Score II; SBP, systolic blood pressure; SOFA, sequential organ failure assessment; WBC, white blood cell count.

### 2.2 Primary outcome

In the American MIMIC‐IV cohort, 2010 patients in total (22.1%) experienced 28‐day mortality, with the highest mortality observed in quintile 5, comprising 391 cases (27.8%; Table 1). Similarly, in the Chinese institutional cohort, there were 276 cases (16.0%) of 28‐day mortality, with the highest mortality observed in group 5, consisting of 75 cases (20.0%). Both cohorts showed significantly elevated serum magnesium in the nonsurvivor group relative to the survivor group (MIMIC‐IV cohort: 0.91 mmol/L vs. 0.86 mmol/L, *p* < 0.001; institutional cohort: 0.92 mmol/L vs. 0.87 mmol/L, *p* = 0.004).

**FIGURE 2:**
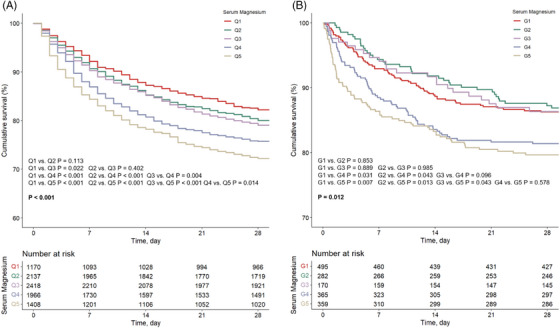
(A, B) Kaplan–Meier survival analysis curves of the two cohorts. Kaplan–Meier curves showing the cumulative probability of all‐cause mortality according to groups within 28 days for the Medical Information Mart for Intensive Care‐IV (MIMIC‐IV) cohort (A) and the institutional cohort (B).

Kaplan–Meier survival analysis results for the two cohorts within 28 days are presented in Figure 2. Both the two cohorts displayed significant divergence in survival probabilities for the five patient groups (both *p* < 0.05). In the American MIMIC‐IV cohort, the highest survival rate was exhibited in Q1, while the lowest survival rate was observed in Q5. However, in Chinese institutional cohort, the highest survival rate was observed in G2 and G3, while the G4 and G5 had similar lowest survival rates.

Univariate logistic regression analysis for the risk of 28‐day all‐cause mortality in the two cohorts were showed in Figure S1. In both cohorts, elevated serum magnesium levels were found to be considerably linked with increased 28‐day all‐cause mortality risk [MIMIC‐IV cohort: odds ratio (OR) = 1.97, 95% confidence interval (CI): 1.53–2.55, *p* < 0.001; Chinese institutional cohort: OR = 2.00, 95% CI: 1.16–3.46, *p* = 0.013].

**FIGURE 3:**
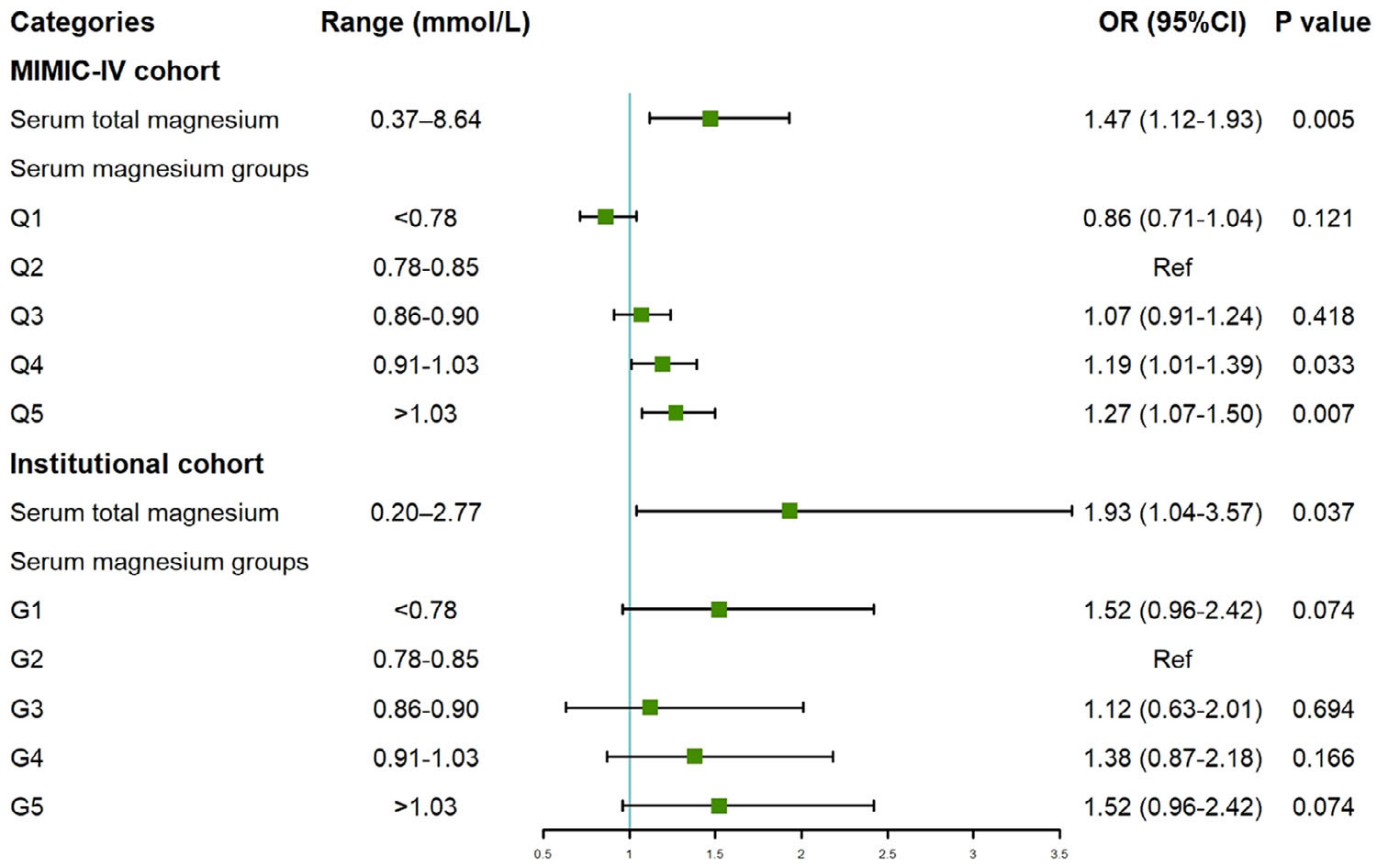
Forest plots of odds ratios (ORs) for the 28‐day mortality in different groups categorized by serum magnesium. In Medical Information Mart for Intensive Care‐IV (MIMIC‐IV) cohort, the *p* for trend among serum magnesium groups is less than 0.001, while in institutional cohort it is 0.028. Institutional cohorts adjusted for age, gender, ethnicity, body mass index (BMI), laboratory tests (white blood cell count, hemoglobin, platelets, serum creatinine, international normalized ratio, serum potassium, and total bilirubin), and comorbidities (hypertension, liver disease, renal failure, diabetes, malignant cancer, chronic pulmonary disease, and cerebrovascular disease). MIMIC‐IV cohort lacked BMI and total bilirubin, while other confounders were the same as the institutional cohort. CI, confidence interval; Ref, reference.

Based on the findings of the univariate logistic regression analysis and considering relevant prognostic variables obtained from clinical experience, the multivariable logistic regression analysis was performed. The findings, presented in Table S3 for the MIMIC‐IV cohort and Table S4 for the Chinese institutional cohort, revealed that patients in the higher serum magnesium groups (MIMIC‐IV cohort: Q4, Q5; Chinese institutional cohort: G4, G5) exhibited a noticeably increased risk of 28‐day mortality, relative to the reference (Q2, G2). After adjusting for confounders, the MIMIC‐IV cohort demonstrated a significant correlation between high serum magnesium (as a continuous variable) and the risk of 28‐day mortality (OR = 1.47, 95% CI: 1.12–1.93, *p* = 0.005). Furthermore, patients in the higher serum magnesium groups (Q4, Q5) exhibited a higher risk of 28‐day mortality (Q4: OR = 1.19, 95% CI: 1.01–1.39, *p* = 0.033; Q5: OR = 1.27, 95% CI: 1.07–1.50, *p* = 0.007), compared to the Q2 group (Figure 3). Similar findings were exhibited in the Chinese institutional cohort. Each unit increase in the serum magnesium index was correlated with a 93% increased risk of 28‐day mortality (OR = 1.93, 95% CI: 1.04–3.57, *p* = 0.037). Patients with higher serum magnesium level (groups 4 and 5) also exhibited a higher risk of 28‐day mortality (G4: OR = 1.38, 95% CI: 0.87–2.18, *p* = 0.166; G5: OR = 1.52, 95% CI: 0.96–2.42, *p* = 0.074; Figure 3).

**FIGURE 4:**
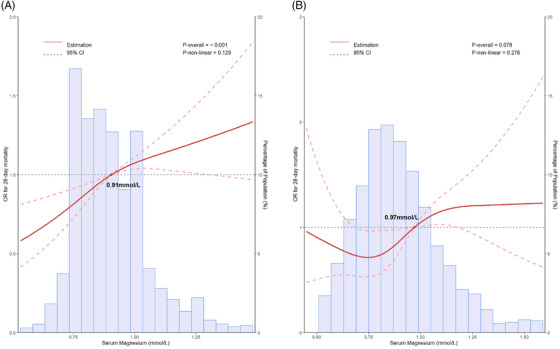
(A, B) Restricted cubic spline curve for the serum magnesium odds ratio (OR) for 28‐day mortality. Association of serum magnesium and 28‐day mortality among US (A) and Chinese (B) patients with sepsis. Institutional cohorts adjusted for age, gender, ethnicity, body mass index (BMI), laboratory tests (white blood cell count, hemoglobin, platelets, serum creatinine, international normalized ratio, serum potassium, and total bilirubin), and comorbidities (hypertension, liver disease, renal failure, diabetes, malignant cancer, chronic pulmonary disease, and cerebrovascular disease). Medical Information Mart for Intensive Care‐IV (MIMIC‐IV) cohort lacked BMI and total bilirubin, while other confounders were the same as the institutional cohort. CI, confidence interval.

The restricted cubic spline (RCS) curves utilized the cutoff value derived from the receiver operating characteristic (ROC) curve as reference (Figure S2). In MIMIC‐IV cohort, the RCS analysis revealed a positive correlation between elevated serum magnesium and OR for 28‐day mortality (*p* overall <0.001), with the cutoff value of 0.91 mmol/L (Figure 4). While a J‐shaped correlation was exhibited between serum magnesium and 28‐day mortality in Chinese institutional cohort (*p* overall = 0.078), with the cutoff value of 0.97 mmol (Figure 4). However, the correlation between serum magnesium and mortality in both cohorts did not show significant nonlinearity (*p* = 0.129 and *p* = 0.278 for nonlinearity, respectively).

### 2.3 Secondary outcomes

The secondary outcomes among the five groups in the MIMIC‐IV cohort are showed in Table 1. Compared to patients with the lower serum magnesium, those in the higher serum magnesium groups exhibited longer ICU length of stay (2.9 vs. 2.9 vs. 2.9 vs. 3.2 vs. 3.4 days, *p* < 0.001), longer hospital length of stay (7.8 vs. 8.0 vs. 8.5 vs. 8.6 vs. 9.2 days, *p* < 0.001), higher 90‐day mortality (25.9% vs. 27.6% vs. 27.5% vs. 31.1% vs. 34.7%, *p* < 0.001), higher ICU mortality (8.1% vs. 10.0% vs. 11.5% vs. 14.8% vs. 17.5%, *p* < 0.001), and higher hospital mortality (13.2% vs. 15.6% vs. 16.7% vs. 19.7% vs. 23.7%, *p* < 0.001).

In terms of 90‐day mortality, ICU mortality and in‐hospital mortality in Chinese institutional cohort, no statistically differences were observed among the five groups (Table S1). However, compare to G2, both G4 and G5 showed higher 90‐day mortality (G4 vs. G2: 19.2% vs. 15.4%; G5 vs. G2: 20.5% vs. 15.4%), ICU mortality (G4 vs. G2: 12.6% vs. 8.6%; G5 vs. G2: 12.3% vs. 8.6%), and in‐hospital mortality (G4 vs. G2: 19.5% vs. 15.8%; G5 vs. G2: 20.8% vs. 15.8%). Patients in the group 2 had obviously longer ICU and hospital length of stay compared to the other four groups.

### 2.4 Subgroup analyses

**FIGURE 5:**
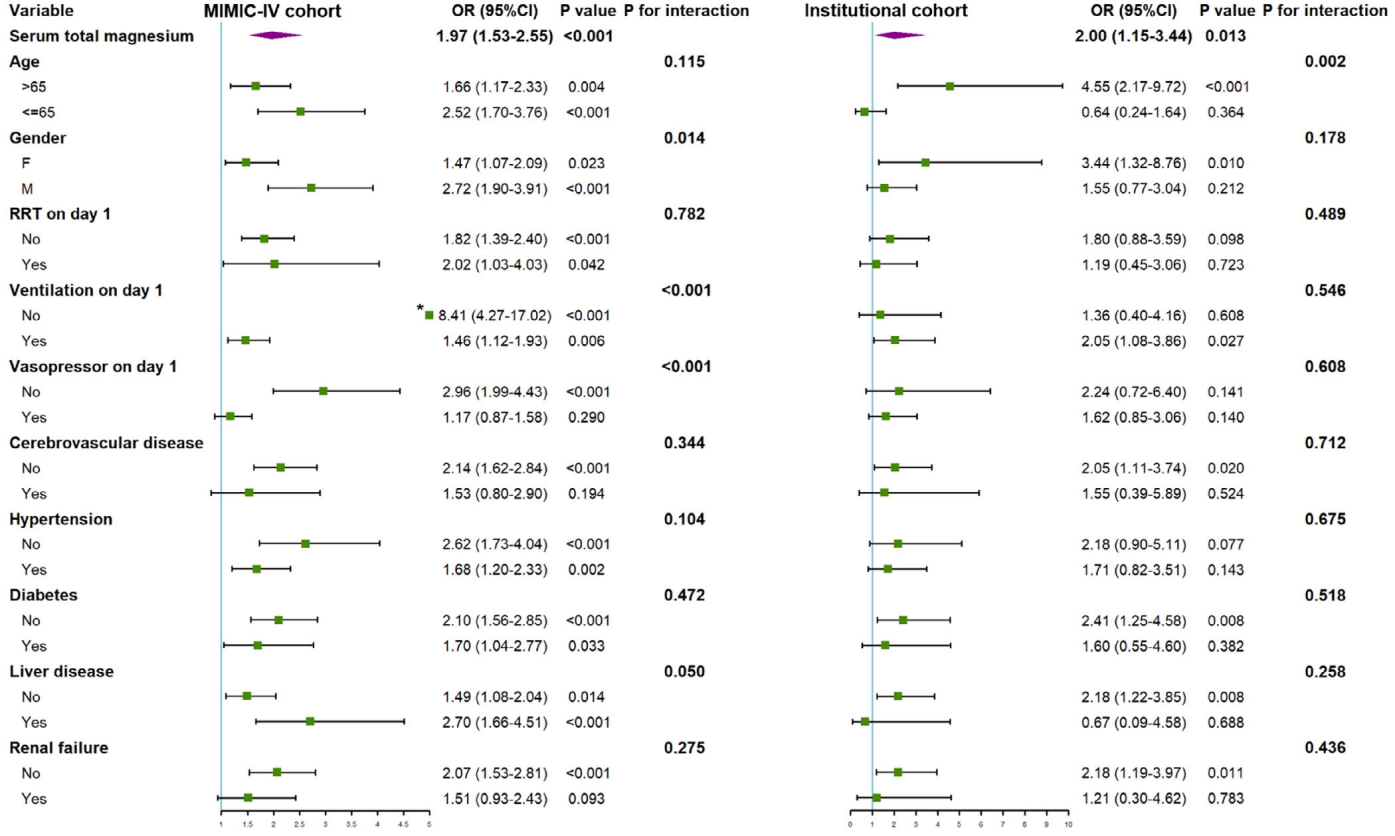
Forest plots of odds ratios (OR) for the 28‐day mortality in different subgroups. *Large data point (exceeds plot limits). CI, confidence interval; MIMIC‐IV, Medical Information Mart for Intensive Care‐IV; RRT, renal replacement therapy.

Further analysis was performed to examine the risk stratification value of serum magnesium as a continuous variable for 28‐day mortality across various subgroups of enrolled patients. These subgroups including age, gender, day 1 interventions [renal replacement therapy (RRT), mechanical ventilation, vasopressors], cerebrovascular disease, hypertension, diabetes, liver disease, and renal failure (Figure 5). In both cohorts, statistically significant interactions were observed in the following four subgroups: (1) female versus male in the MIMIC‐IV cohort [OR 1.47 (1.07–2.09) vs. 2.72 (1.90–3.91), *p* = 0.014 for interaction], (2) patients aged >65 versus ≤65 years old in the institutional cohort [OR 4.55 (2.17–9.72) vs. 0.64 (0.24–1.64), *p* = 0.002 for interaction], (3) patients without versus with the need for mechanical ventilation on ICU day 1 in the MIMIC‐IV cohort [OR 8.41 (4.27–17.02) vs. 1.46 (1.12–1.93), *p* < 0.001 for interaction], and (4) patients without versus with the need for vasopressor on ICU day 1 in the MIMIC‐IV cohort [OR 2.96 (1.99–4.43) vs. 1.17 (0.87–1.58), *p* < 0.001 for interaction].

## 3 DISCUSSION

The findings of the current study showed a significant association between serum magnesium levels at the onset of sepsis and 28‐day all‐cause mortality in critically ill patients. This association remained consistent across various subgroups. Furthermore, elevated serum magnesium levels were also linked to increased 90‐day mortality, ICU mortality, and in‐hospital mortality. These findings suggest that serum magnesium at the onset of sepsis could be a practical parameter for risk stratification and potentially act as an independent risk factor for septic patients.

Previous study reported that higher serum magnesium levels at ICU admission were associated with increased in‐hospital mortality in patients with sepsis,^22^ which is consistent with our findings. Xia et al. mainly explored the trajectory of magnesium levels in septic patients during ICU stay and its relationship with in‐hospital mortality, providing important evidence for clinical management. However, the study did not clarify the temporal relationship between magnesium and sepsis course, and was limited to a single center. Our study focused on serum magnesium levels at the onset of sepsis, utilizing two international cohorts covering a large number of samples, and demonstrated that serum magnesium at the onset of sepsis was a valuable marker for patient risk stratification. The utilization of the MIMIC database, which encompasses a large number of septic patients in the ICU, enabled the evaluation of the relationship between serum magnesium levels and mortality in this population. The Chinese institutional cohort, derived from real‐world data from the ICU of a university hospital in China, was employed to further validate this relationship. The consistent results obtained from these two cohorts validate the relationship between high serum magnesium levels and increased mortality in sepsis across different ethnicities and medical conditions. These findings make the results applicable to at least North American and East Asian populations.

Assessment of serum magnesium concentration is a commonly practice in clinical practice to evaluate magnesium status, with the range considered normal generally falling between 0.70 and 0.99 mmol/L.^23^ Previous studies have consistently shown a significantly higher incidence of hypomagnesemia compared to hypermagnesemia in medical settings.^24^, ^25^ In the context of severe sepsis, hypomagnesemia is frequently observed.^26^ In line with this, our study found that the median serum magnesium level in septic patients was 0.86 mmol/L (IQR: 0.82–0.98) in the MIMIC‐IV cohort and 0.87 mmol/L (IQR: 0.76–1.01) in Chinese institutional cohort. Furthermore, the percentage of individuals in the Q4–Q5 group was 37.1% in MIMIC‐IV cohort, and 43.7% in Chinese institutional cohort. These findings suggest that, at the onset of sepsis, high magnesium concentrations may be more prevalent than low magnesium concentrations.

Previous studies have explored the correlation between serum magnesium levels and the risk of incidence and mortality in various diseases. For instance, Tan et al. found that critically ill patients with acute myocardial infarction who had high‐normal serum magnesium levels or hypermagnesemia tended to have a higher 30‐day in‐hospital mortality rate.^27^ Sharma et al. reported that a high prevalence hypermagnesemia in patients with COVID‐19 was correlated with an elevated risk of mortality.^20^ Lancaster et al. found that higher magnesium levels were linked to an increased risk of atrial fibrillation following cardiac surgery.^28^ Additionally, a study involving 39,193 participants showed that both hypomagnesemia and hypermagnesemia at discharge were associated with higher 1‐year mortality.^29^ In critically ill patients, serum magnesium levels were strongly associated with mortality.^30^ Despite extensive research on the effects of hypomagnesemia, there is a paucity of studies investigating the effects of hypermagnesemia and its role in mortality. Recent studies on critically ill patients have showed that serum magnesium concentrations exceed 2.4 mg/dL, 2.5 mg/dL, and 2.6 mg/dL significantly increase the risk of death is significantly increased.^31^, ^32^, ^33^ Our findings were consistent with the results of the previous study and provide further support for the association between high serum magnesium levels and a poor prognosis.

Serum magnesium plays an essential role in the pathophysiology of sepsis. As the second most abundant intracellular cation, magnesium participates in essential physiological processes, including blood pressure regulation, neuronal transmission, muscle contraction, and immune regulation.^34^, ^35^ Several factors contribute to the correlation between high serum magnesium levels and an unfavorable prognosis in septic patients. First, from a cardiovascular perspective, elevated serum magnesium induces vasodilation and lowers blood pressure by affecting vascular tone and the renin–angiotensin–aldosterone system.^36^ Our study demonstrated that patients with high serum magnesium exhibited lower mean arterial pressure, higher lactate levels, and an increased requirement for vasopressor. Considering the often unstable cardiovascular dynamics observed in septic patients,^37^ the exacerbation of this instability by high magnesium levels may contribute to poor outcomes. Additionally, magnesium can impede cardiac potassium channels, leading to malignant arrhythmias and heart failure,^23^ further increasing the risk of mortality. Previous study has indicated racial disparities in cardiovascular responses.^38^ Therefore, discrepancies in magnesium metabolism and cardiovascular responses among racial differences may explain the variations observed in the results of the two cohorts in our study. Second, in terms of respiratory function, elevated magnesium levels impede neuromuscular transmission, resulting muscle paralysis, including the respiratory muscles and diaphragm, thereby impairing respiratory function and contributing to respiratory failure.^39^ Our study demonstrated that among patients who did not require mechanical ventilation on ICU day 1 in the MIMIC‐IV cohort, each unit increase in serum magnesium was correlated with a 7.41‐fold increase in the risk of 28‐day mortality. Third, magnesium is essential for optimal immune function and the regulation of inflammation.^40^ High levels of magnesium can impair the function of endotoxin‐stimulated spleen lymphocytes.^41^ Elevated serum magnesium levels may disrupt normal immune responses and exacerbate the dysregulated inflammatory state in sepsis, subsequently leading to poorer prognosis.

Although there was a similarity in the primary outcome regarding the correlation between high serum magnesium and increased 28‐day mortality in septic patients, there were still divergent results observed in the two cohorts. The distribution of mortality across various groups differed between the MIMIC‐IV cohort and the institutional cohort. Moreover, the RCS analysis revealed a positive association between increasing serum magnesium levels and the OR for 28‐day mortality in the MIMIC‐IV cohort, whereas the Chinese institutional cohort exhibited a J‐shaped correlation. Several factors may account for these differences, including: (1) Baseline variations between the cohorts, including ethnicity (the majority of the MIMIC‐IV cohort consisted of White individuals, while the Chinese institutional cohort primarily comprised East‐Asian patients), SOFA score (3 in the MIMIC‐IV and 5 in the Chinese institutional cohort), and interventions (a higher percentage of ventilation in the MIMIC‐IV cohort and a higher percentage of RRT and vasopressors in the Chinese institutional cohort). (2) Differences in the distribution of serum magnesium. The normal range for serum magnesium is 0.70–0.99 mmol/L, and the bar chart in Figure 4 indicated a higher proportion of patients with hypomagnesemia in the Chinese institutional cohort, compared to the MIMIC‐IV cohort. Within the Chinese institutional cohort, the group with lower serum magnesium levels (G1) exhibited higher 28‐day mortality than the group with serum magnesium within the normal range (G2), suggesting that hypomagnesemia and its associated poor prognosis may contribute to the descending trend observed on the left side of the RCS curve in the Chinese institutional cohort. However, in multiple multivariable logistic regression models, the difference between the G1 and G2 did not reach statistical significance.

Subgroup analysis revealed that serum magnesium levels might have predictive value in specifically male septic patients, potentially due to the influence of estrogen on females. Estrogen can affect magnesium distribution, enhance magnesium utilization, and promote its uptake in soft tissue and bone.^42^ Additionally, the study observed a lower proportion of women among patients with higher serum magnesium levels, thus providing additional support for this observation. Furthermore, monitoring serum magnesium levels may yield greater prognostic benefits for elderly patients or those who do not require mechanical ventilation or vasopressors upon ICU admission.

The present study has several limitations that should be acknowledged. First, its retrospective design precludes the establishment of a causal relationship. Second, the study solely collected serum magnesium levels, which may not fully represent the overall magnesium status in the body. Factors such as intracellular magnesium levels were not assessed. Third, the precise mechanism underlying the association between high serum magnesium levels and poor prognosis in sepsis remains unknown. Fourth, in the MIMIC‐IV cohort, there was an imbalance in the distribution of both known and unknown risk factors among the different groups, potentially attributing the higher mortality observed in patients with higher serum magnesium levels to their greater burden of acute illness and comorbidities. Although significant statistical differences observed when comparing baseline data between groups, this may be attributable to the larger sample size. However, it is important to note that the normal concentration range of serum magnesium is narrow (0.70–0.99 mmol/L), and even slight changes in concentration can potentially impact patients. Therefore, to ensure the reliability of the results, the study categorized serum magnesium into quintiles and extensively adjusted for the results of the univariate analysis. The association between high serum magnesium levels and increased mortality remained significant. Last, subsequent serum magnesium levels during ICU hospitalization were not collected, limiting the analysis of dynamic magnesium fluctuations and their impact on prognosis. Therefore, cautious interpretation of the findings is warranted, despite the observed differences in mortality. Further research is necessary to better comprehend the relationship between serum magnesium levels and mortality in sepsis, as well as to investigate the potential effects of magnesium fluctuations on patient outcomes.

## 4 METHODS

### 4.1 Data source

This research made use of data from the MIMIC‐IV database (version 2.2) and a Chinese institutional cohort.^43^ The MIMIC‐IV dataset encompasses extensive, high‐fidelity electronic health information for over 70,000 individuals treated in the ICU at Beth Israel Deaconess Medical Center, Boston, Massachusetts, covering the period from 2008 to 2019. To safeguard patient privacy, all personal identifiers were removed, and patient identities were replaced with random codes, negating the requirement for patient consent and ethical approval. Author Le Li (certification number 50858519) acquired permission for accessing the database and performed the extraction of data. The institutional critical care cohort was collected in the ICU of Zhongnan Hospital, Wuhan University, a university‐affiliated hospital with 66 ICU beds. The hospital is a highly respected and renowned large‐scale medical institution in China, with its Intensive Care Medicine department being particularly esteemed and influential nationwide. Researchers meticulously reviewed medical records from this ICU, covering the period from January 2018 to December 2023.

### 4.2 Patient selection

The criteria for patient enrollment in this study consisted of adult patients admitted to the ICU with sepsis, as recognized by the Sepsis 3.0 definition (Figure S3). In cases of multiple ICU admissions by patients, only data from their first ICU stay were analyzed. Exclusion was based on criteria such as: (1) ICU stays of under 24 h; (2) treatment with magnesium persulfate within 72 h after ICU admission; and (3) absence of serum magnesium data.

### 4.3 Data extraction and management

Data were obtained from the MIMIC‐IV database through structured query language (SQL) queries, with the SQL script codes offered on GitHub (https://github.com/MIT‐LCP/mimic‐iv). The collected data included baseline demographic information, the maximum serum magnesium level at the onset of sepsis, treatments administered within 24 h of ICU admission, the records at the onset of sepsis (relevant clinical scores, vital signs, laboratory tests), and comorbidities.

To mitigate potential bias, the analysis excluded variables where the missing value was above 20%. For those with lower missing data levels, variables were subjected to multiple imputation using a random forest algorithm, which was implemented through the “mice” package in R (version R.4.3.1).^44^ The imputation procedure utilized nonmissing variables as predictors. The missingness for each variable is presented in Figure S4.

In the institutional cohort, patient data from the hospital's medical record system were obtained, including demographic characteristics, clinical variables, and discharge status. The data collection process for this cohort mirrored that of the MIMIC‐IV cohort. However, there were differences in the relevant clinical scores, with only the SOFA score and the Acute Physiology and Chronic Health Evaluation II (APACHE II) score being recorded. Additionally, laboratory indicators such as interleukin‐6 and procalcitonin were included. Detailed information regarding the specific variables extracted can be found in the Supporting Information.

### 4.4 Outcomes

The primary outcome was all‐cause 28‐day mortality. Secondary outcomes included ICU, hospital and 90‐day mortality rates, ICU and hospital length of stay.

### 4.5 Statistical analysis

The Kolmogorov–Smirnov test was employed to assess the normality of continuous variables. Continuous variables with a normal distribution were noted as mean ± standard deviation, and those skewed as median (IQR). Categorical variables were represented by numbers and percentages. Patients in both cohorts were divided into five groups, categorized by the serum magnesium quintiles from the MIMIC‐IV dataset. Normal distribution variables were analyzed using one‐way ANOVA, while skewed distribution variables were assessed with the Kruskal–Wallis *H*‐test. For the analysis of categorical variables, the Pearson's χ^2^ test or Fisher's test was employed as needed. Univariate logistic regression analysis was employed to assess the relationship between each characteristic and 28‐day all‐cause mortality. The association between serum magnesium levels and 28‐day all‐cause mortality was evaluated by multivariable logistic regression models. To mitigate the potential of excessive adjustment for potential confounders, a stepwise approach with four levels of adjustment was employed. Model 1 included adjustments for age, gender, body mass index (BMI), and ethnicity. Model 2 incorporated these factors plus the SOFA score. Model 3 built upon Model 1 with the addition of laboratory tests (white blood cell count, hemoglobin, platelets, serum creatinine, international normalized ratio, serum potassium, and total bilirubin). Model 4 further incorporated the confounders from Model 3 as well as comorbidities (hypertension, liver disease, renal failure, diabetes, malignant cancer, chronic pulmonary disease, and cerebrovascular disease). Moreover, the MIMIC‐IV cohort lacked BMI and total bilirubin, while other confounders remained the same as described above.

Furthermore, RCS plots were employed to analyze the correlation between serum magnesium levels and 28‐day all‐cause mortality. Survival curves were constructed with the Kaplan–Meier method and compared using the log‐rank test across the five groups. In cases where the curves crossed, the Tarone–Ware test was employed.

Subgroup analysis was conducted to explore the potential association of serum magnesium as a continuous variable in different subgroups based on age, gender, the demand for interventions (RRT, mechanical ventilation, and vasopressor) on the day 1, cerebrovascular disease, hypertension, liver disease, renal failure, and diabetes.

All statistical analyses were conducted using the R package (version 4.3.1) and IBM SPSS Statistics (version 26). Statistical significance was concluded for *p* values below 0.05 (two‐tailed).

## 5 CONCLUSION

Higher serum magnesium levels upon sepsis onset are independently associated with increased mortality. Serum magnesium can serve as a predictive factor for the risk of short‐term mortality in septic patients. Monitoring serum magnesium levels may provide valuable insights for clinical decision making and facilitate the implementation of effective disease management strategies.

## AUTHOR CONTRIBUTIONS

Le Li, Bo Hu, and Chang Hu designed this study and protocol development; Le Li and Qiuyue Zhao were responsible for the data collection; Le Li, Xiao Liu, and Yaohui Liu were responsible for data analysis; Le Li and Li Li conducted the manuscript writing; Kailin Guo, Dongsu Zhang, Chang Hu, and Bo Hu critically revised the manuscript; Le Li, Li Li, Chang Hu, and Bo Hu provided final approval for this version to be published. All the authors agreed to be accountable for all aspects of the work in ensuring that questions related to the accuracy or integrity of any part of the work are appropriately investigated and resolved. All the authors have read and approved the final manuscript.

## CONFLICT OF INTEREST STATEMENT

The authors declare no conflicts of interest.

## ETHICS STATEMENT

The establishment of MIMIC‐IV database was approved by the Massachusetts Institute of Technology (Cambridge, MA) and Beth Israel Deaconess Medical Center (Boston, MA), and consent was obtained for the original data collection. Therefore, the ethical approval statement and the need for informed consent were waived for this manuscript. This retrospective study was approved by the Clinical/Scientific Project of the Medical Ethics Committee, Zhongnan Hospital of Wuhan University, registration number 2024110K. This study took a retrospective approach, utilizing patient information from the medical record system. Given the minimal risk involved, informed consent was waived by the patients.

## REFERENCES

## Supporting information

Supporting Information

## Data Availability

The datasets generated during and/or analyzed during the current study are available from the corresponding author on reasonable request.
