# Supplementary material for: High serum magnesium level is associated with increased mortality in patients with sepsis: an international, multicenter retrospective study
Source: MedComm (2020). 2024 Sep 17;5(10):e713. doi: 10.1002/mco2.713 (PMC11406045; doi:10.1002/mco2.713)
Supplement: Supplementary file 1 — Supporting Information [file MCO2-5-e713-s001.docx]

**High Serum Magnesium Level is Associated with Increased Mortality in Patients with Sepsis: An International, Multi-Center Retrospective Study**

**The short running title:** Serum Magnesium and Sepsis Mortality

Le Li ^1, 2#^, Li Li ^1, 2#^, Qiuyue Zhao ^1, 2^, Xiao Liu ^1, 2^, Yaohui Liu ^1, 2^, Kailin Guo ^1, 2^, Dongsu Zhang ^1, 2^, Chang Hu ^1, 2^*, Bo Hu ^1, 2^*

^1^ Department of Critical Care Medicine, Zhongnan Hospital of Wuhan University, Wuhan 430071, Hubei, China

^2^ Clinical Research Center of Hubei Critical Care Medicine, Wuhan 430071, Hubei, China

# Le Li and Li Li contribute equally to the article.

**Correspondence to:** Bo Hu, MD; Zhongnan Hospital of Wuhan University; Wuhan 430071, Hubei, China; Email: [hubozn@whu.edu.cn](mailto:hubozn@whu.edu.cn)

**And Correspondence to:** Chang Hu, MD; Zhongnan Hospital of Wuhan University; Wuhan 430071, Hubei, China; Email: [huchang@whu.edu.cn](mailto:huchang@whu.edu.cn)

**Acknowledgements:** We would like to express our gratitude to Dr. Lu Li for her valuable assistance in data extraction.

Supplementary Tables and Figures cited in main text

- Variables extraction for two cohorts
- Table S1: Characteristics and outcomes of participants categorized by serum magnesium in the Chinese institutional cohort
- Table S2: Characteristics and outcomes of participants categorized by cohorts
- Table S3: Association of serum magnesium and 28-day mortality by multivariable-adjusted logistic analysis in the American MIMIC-IV cohort
- Table S4: Association of serum magnesium and 28-day mortality by multivariable-adjusted logistic analysis in the Chinese institutional cohort
- Figure S1: Univariate analysis of risk factors associated with 28-day mortality in patients with sepsis
- Figure S2: Receiver operating characteristic curve of serum magnesium for sepsis
- Figure S3: Diagnostic criteria for sepsis
- Figure S4: Percentage of missing data of variable in MIMIC-IV cohort

**Variables extraction for two cohorts**

For the MIMIC-IV database, we collected baseline demographic information, including age, gender, ethnicity, and body mass index (BMI). The maximum serum magnesium level at the onset of sepsis was documented, along with treatments administered within 24 hours of ICU admission, such as mechanical ventilation, vasopressors, and renal replacement therapy (RRT). Vasopressor use was defined as the administration of norepinephrine, epinephrine, dopamine, dobutamine, milrinone, or vasopressin. Information regarding comorbidities, such as chronic obstructive pulmonary disease (COPD), hypertension, liver disease, renal failure, cerebrovascular disease, diabetes and malignant cancer, was extracted based on the International Classification of Diseases coding system. Additionally, initial records at the onset of sepsis were collected, including severity of illness scores [sequential organ failure assessment (SOFA), simplified acute physiology score II (SAPSII), acute physiology and chronic health evaluation III (APSIII), overall acute severity of Illness score (OASIS), Glasgow coma scale (GCS)], vital signs [heart rate, systolic blood pressure (SBP), diastolic blood pressure (DBP), mean arterial pressure (MAP), respiratory rate, body temperature], as well as laboratory tests [creatinine (Cr), blood urea nitrogen (BUN), white blood cell count (WBC), hemoglobin, platelets (PLT), prothrombin time (PT), partial thromboplastin time (PTT), international normalized ratio (INR), alanine aminotransferase (ALT), aspartate aminotransferase (AST), total bilirubin, potassium level, chloride level, sodium level, calcium level, phosphate level, glucose level, and lactate level.

In institutional cohort, the in-hospital patient data was obtained from the medical record system, which included records of demographic characteristics, clinical variables, and discharge status. The data collection for this cohort was similar to the MIMIC-IV cohort, including baseline demographic information, vital signs, treatments administered within 24 hours of ICU admission, and comorbidities. However, there was a difference in the severity of illness scores, as only the SOFA score and the Acute Physiology and Chronic Health Evaluation II (APACHE II) score were recorded. Additionally, laboratory indicators such as interleukin-6 and procalcitonin were included.

**Table S1. Characteristics and outcomes of participants categorized by Serum magnesium in the institutional cohort**

| Characteristics | Total | Serum total magnesium (mmol/L) | | | | | | | P-value |  |
| --- | --- | --- | --- | --- | --- | --- | --- | --- | --- | --- |
|  | (0.20–2.77) | G1 (<0.78) | G2 (0.78-0.85) | | G3 (0.86-0.90) | | G4 (0.91-1.03) | G5 (>1.03) |  |  |
| Number of patients, n | 1727 | 500 | 292 | 180 | | 380 | | 375 |  |  |
| **Baseline variables** |  |  |  |  | |  | |  |  |  |
| Age(year) | 65.0 (54.0-74.0) | 65.0 (54.0-74.0) | 64.5 (54.0-75.0) | 65.0 (56.8-75.0) | | 65 (55-74) | | 64 (53-75) | 0.783 |  |
| BMI (kg/m^2^) | 22.6 (20.2-24.5) | 22.7 (20.3-24.5) | 22.5 (19.6-24.8) | 22.5 (20.2-24.5) | | 22.9 (20.3-24.5) | | 22.8 (20.3-24.7) | 0.890 |  |
| Female | 577 (33.4) | 196 (39.2) | 98 (33.6) | 60 (33.3) | | 107 (28.2) | | 116 (30.9) | 0.002 |  |
| **Vital signs** |  |  |  |  | |  | |  |  |  |
| Heart rate (b/min) | 94 (82-107) | 95 (83-108) | 93 (80-106) | 93 (82-105) | | 95 (83-107) | | 95 (83-108) | 0.427 |  |
| SBP (mmHg) | 86 (73-98) | 87 (75-99) | 87 (76-100) | 86 (74-98) | | 86 (70-99) | | 85 (71-97) | 0.156 |  |
| DBP (mmHg) | 46 (39-52) | 46 (41-53) | 46 (40-53) | 45 (39-52) | | 45 (39-52) | | 45 (37-51) | 0.030 |  |
| MAP (mmHg) | 59 (49-67) | 60 (50-67) | 59 (50-69) | 59 (49-66) | | 58 (48-67) | | 58 (46-66) | 0.328 |  |
| Respiratory rate (b/min) | 18 (16-21) | 18 (16-21) | 19 (17-21) | 19 (16-22) | | 19 (17-21) | | 19 (16-21) | 0.838 |  |
| Temperature (℃) | 37.5 (36.9-38.3) | 37.5 (37.0-38.4) | 37.5 (37.0-38.3) | 37.6 (36.9-38.3) | | 37.5 (36.9-38.4) | | 37.5 (36.8-38.3) | 0.362 |  |
| **Laboratory parameters** | |  |  |  | |  | |  |  |  |
| AST/ALT | 1.9 (1.3-2.9) | 1.9 (1.4-2.8) | 1.8 (1.3-2.8) | 1.9 (1.3-2.8) | | 2.0 (1.3-3.1) | | 1.9 (1.4-3.0) | 0.694 |  |
| IL-6 (pg/mL) | 213 (57-878) | 301 (72-989) | 207 (62-802) | 268 (69-1294) | | 179 (54-710) | | 171 (41-774) | 0.004 |  |
| Procalcitonin (ng/mL) | 7.2 (1.2-36.5) | 8.8 (1.3-35.5) | 4.6 (0.9-23.9) | 6.9 (1.1-36.9) | | 6.1 (0.9-35.3) | | 11.4 (2.0-43.3) | 0.008 |  |
| Cr (umol/L) | 131.5 (80.4-260.6) | 109.0 (74.0-204.3) | 108.9 (71.4-228.2) | 116.9 (75.5-228.6) | | 146.4 (84.9-280.1) | | 208.3 (112.7-364.8) | <0.001 |  |
| Total bilirubin (umol/L) | 22.0 (14.1-44.4) | 22.0 (14.0-44.5) | 23.0 (14.8-39.9) | 20.5 (13.5-32.4) | | 24.4 (14.2-48.7) | | 22.2 (13.8-46.7) | 0.585 |  |
| WBC (K/uL) | 13.7 (9.1-20.3) | 13.2 (8.6-19.7) | 14.0 (9.6-20.8) | | 14.3 (10.0-20.0) | | 14.2 (9.2-20.3) | 13.8 (9.3-20.5) | 0.551 |  |
| Hemoglobin (g/L) | 109 (90-127) | 109 (89-127) | 108 (90-124) | | 108 (88-126) | | 108 (87-126) | 113 (93-139) | 0.215 |  |
| Platelets (K/uL) | 167 (97-245) | 168 (105-246) | 176 (107-243) | | 169 (95-274) | | 164 (95-241) | 161 (93-234) | 0.508 |  |
| PT (s) | 15.3 (13.2-19.0) | 15.6 (13.5-19.0) | 14.9 (13.0-17.7) | | 14.6 (12.6-18.0) | | 15.5 (13.1-19.5) | 15.3 (13.2-20.4) | 0.005 |  |
| APTT (s) | 35.2 (30.4-44.5) | 34.6 (30.3-41.6) | 34.1 (29.5-42.1) | | 34.1 (29.5-42.9) | | 35.5 (30.8-45.1) | 37.6 (31.6-51.4) | ＜0.001 |  |
| INR | 1.4 (1.2-1.7) | 1.4 (1.2-1.7) | 1.4 (1.2-1.6) | | 1.3 (1.2-1.6) | | 1.4 (1.2-1.8) | 1.4 (1.2-1.9) | 0.005 |  |
| Lactate (mmol/L) | 332 (226-534) | 280 (207-428) | 336 (234-531) | | 324 (219-519) | | 367 (240-660) | 383 (241-642) | ＜0.001 |  |
| Potassium (mmol/L) | 4,4 (4.0-4.9) | 4.3 (3.9-4.7) | 4.3 (3.9-4.8) | | 4.5 (4.0-5.0) | | 4.5 (4.1-5.1) | 4.5 (4.1-5.2) | ＜0.001 |  |
| Chloride (mmol/L) | 108 (104-112) | 109 (104-112) | 108 (104-111) | | 107 (103-112) | | 108 (104-112) | 108 (103-113) | 0.380 |  |
| Sodium (mmol/L) | 146 (141-152) | 144 (140-149) | 144 (140-149) | | 145 (140-150) | | 147 (141-153) | 150 (144-157) | 0.000 |  |
| Calcium (mmol/L) | 2.1 (2.0-2.3) | 2.1 (2.0-2.2) | 2.1 (2.0-2.2) | | 2.1 (2.0-2.2) | | 2.1 (2.0-2.3) | 2.2 (2.0-2.3) | 0.003 |  |
| Glucose (mmol/L) | 10.4 (8.2-14.5) | 10.1 (7.9-13.4) | 10.1 (7.8-13.2) | | 10.3 (8.2-14.4) | | 10.9 (8.4-14.9) | 11.4 (8.5-15.8) | ＜0.001 |  |
| **Score system** |  |  |  | |  | |  |  |  |  |
| SOFA | 5 (3-9) | 9 (6-12) | 9 (6-11) | | 9 (6-13) | | 10 (7-13) | 11 (7-14) | ＜0.001 |  |
| APACHE II | 23 (18-30) | 21 (16-27) | 22 (16-28) | | 23 (18-30) | | 25 (19-31) | 26 (21-32) | 0.000 |  |
| **Interventions, n (%)** |  |  |  | |  | |  |  |  |  |
| Ventilation | 1047 (60.6) | 292 (58.4) | 164 (56.2) | | 113 (62.8) | | 236 (62.1) | 242 (64.5) | 0.161 |  |
| RRT | 443 (25.7) | 89 (17.8) | 56 (19.2) | | 43 (23.9) | | 116 (30.3) | 139 (37.1) | ＜0.001 |  |
| Vasopressor | 1012 (58.6) | 272 (54.4) | 163 (55.8) | | 99 (55.0) | | 230 (60.6) | 248 (66.1) | 0.005 |  |
| **Comorbidities, n (%)** |  |  |  | |  | |  |  |  |  |
| COPD | 25 (1.4) | 5 (1.0) | 2 (0.7) | | 1 (0.6) | | 10 (2.6) | 7 (1.9) | 0.182 |  |
| Hypertension | 816 (47.2) | 222 (44.4) | 136 (46.6) | | 80 (44.4) | | 188 (49.5) | 190 (50.7) | 0.345 |  |
| Heart failure | 74 (4.3) | 14 (2.8) | 11 (3.8) | | 3 (1.7) | | 20 (5.3) | 26 (6.9) | 0.026 |  |
| Liver disease | 126 (7.3) | 462 (92.4) | 274 (93.8) | | 172 (95.6) | | 346 (91.1) | 337 (89.9) | 0.078 |  |
| Renal failure | 223 (12.9) | 63 (12.6) | 35 (15.7) | | 14 (7.8) | | 60 (15.8) | 51 (13.6) | 0.174 |  |
| Cerebrovascular disease | 268 (15.5) | 69 (13.8) | 40 (13.7) | | 28 (15.6) | | 65 (17.1) | 66 (17.6) | 0.415 |  |
| Diabetes | 427 (24.7) | 136 (27.2) | 78 (26.7) | | 38 (21.1) | | 88 (23.2) | 87 (23.2) | 0.383 |  |
| Cancer | 293 (17.0) | 97 (19.4) | 55 (18.8) | | 27 (15.0) | | 62 (16.3) | 52 (13.9) | 0.256 |  |
| **Primary outcome** |  |  |  | |  | |  |  |  |  |
| 28-day mortality, n (%) | 276 (16.0) | 69 (13.8) | 39 (13.4) | | 23 (12.8) | | 70 (18.4) | 75 (20.0) | 0.028 |  |
| **Secondary outcome** |  |  |  | |  | |  |  |  |  |
| 90-day mortality, n (%) | 294 (17.0) | 73 (14.6) | 45 (15.4) | | 26 (14.4) | | 73 (19.2) | 77 (20.5) | 0.091 |  |
| ICU mortality, n (%) | 182 (10.5) | 44 (8.8) | 25 (8.6) | | 19 (10.6) | | 48 (12.6) | 46 (12.3) | 0.217 |  |
| In-hospital mortality, n (%) | 303 (17.5) | 77 (15.4) | 46 (15.8) | | 28 (15.6) | | 74 (19.5) | 78 (20.8) | 0.168 |  |
| ICU length of stay, day | 4.0 (1.0-9.0) | 3.0 (1.0-8.0) | 5.0 (2.0-10.8) | | 4.0 (1.3-9.0) | | 4.0 (1.0-10.0) | 4.0 (1.0-9.0) | 0.024 |  |
| Hospital length of stay, day | 15.0 (8.0-27.0) | 15.0 (9.0-26.8) | 17.0 (9.0-30.0) | | 16.0 (7.0-25.0) | | 14.0 (7.0-26.0) | 14.0 (6.0-24.0) | 0.004 |  |

Abbreviation: SBP- systolic blood pressure; DBP- diastolic blood pressure; MAP- mean arterial pressure; ALT - Alanine Aminotransferase; AST- Aspartate Aminotransferase ; Cr- creatinine; WBC- white blood cell count; PT-prothrombin time; APTT- partial thromboplastin time; INR- international normalized ratio; SOFA- Sequential Organ Failure Assessment; APACHE II - Acute Physiology and Chronic Health Evaluation II; RRT- Renal Replacement Therapy; COPD- Chronic Obstructive Pulmonary Disease; ICU - intensive care unit; IL-6 - Interleukin-6.

**Table S2: Characteristics and outcomes of participants categorized by cohorts**

| Characteristics | Total | The MIMIC-IV cohort | | The institutional cohort | P-value | |  |
| --- | --- | --- | --- | --- | --- | --- | --- |
|  | (n=9099) | （n=9099） | | （n=1408） |  |  |  |
| **Baseline variables** |  |  |  | |  |  |  |
| Age(year) | 68.4 (57.2-79.3) | 69.3 (57.9-80.0) | 65.0 (54.0-74.0) | | <0.001 |  |  |
| Female | 4252 (39.3) | 3675 (40.4) | 577 (33.4) | | <0.001 |  |  |
| **Vital signs** |  |  |  | |  |  |  |
| Heart rate (b/min) | 86 (76-98) | 85 (75-96) | 94 (82-107) | | <0.001 |  |  |
| SBP (mmHg) | 111 (101-123) | 114 (105-125) | 86 (73-98) | | <0.001 |  |  |
| DBP (mmHg) | 58 (52-66) | 60 (54-67) | 46 (39-52) | | <0.001 |  |  |
| MAP (mmHg) | 57 (50-64) | 57 (50-64) | 59 (49-67) | | 0.001 |  |  |
| Respiratory rate (b/min) | 19 (17-22) | 19 (17-22) | 18 (16-21) | | <0.001 |  |  |
| Temperature (℃) | 36.9 (36.6-37.3) | 36.8 (36.6-37.2) | 37.5 (36.9-38.3) | | <0.001 |  |  |
| **Laboratory parameters** |  |  |  | |  |  |  |
| Cr (umol/L) | 114.9 (79.6-210.0) | 114.9 (79.6-203.3) | 131.5 (80.4-260.6) | | <0.001 |  |  |
| WBC (K/uL) | 13.7 (9.7-18.9) | 13.7 (9.8-18.7) | 13.7 (9.1-20.3) | | 0.531 |  |  |
| Hemoglobin (g/L) | 99 (83-116) | 97 (82-113) | 109 (90-127) | | <0.001 |  |  |
| Platelets (K/uL) | 168 (112-238) | 168 (114-237) | 167 (97-245) | | 0.097 |  | |
| PT (s) | 14.9 (13.0-18.5) | 14.8 (13.0-18.3) | 15.3 (13.2-19.0) | | <0.001 |  |  |
| INR | 1.3 (1.2-1.7) | 1.3 (1.2-1.7) | | 1.4 (1.2-1.7) | <0.001 | |  |
| Magnesium (mmol/L) | 0.87 (0.81-0.99) | 0.86 (0.82-0.99) | | 0.87 (0.76-1.01) | <0.001 | |  |
| Potassium (mmol/L) | 4.5 (4.1-5.1) | 4.5 (4.2-5.1) | | 4.4 (4.0-4.9) | <0.001 | |  |
| Chloride (mmol/L) | 106 (102-110) | 106 (102-110) | | 108 (104-112) | <0.001 | |  |
| Sodium (mmol/L) | 141 (138-144) | 140 (137-143) | | 146 (141-152) | <0.001 | |  |
| Calcium (mmol/L) | 4.2 (3.9-4.5) | 4.3 (4.1-4.5) | | 2.1 (2.0-2.3) | <0.001 | |  |
| Glucose (mmol/L) | 9.3 (7.4-12.4) | 9.2 (7.3-12.1) | | 10.4 (8.2-14.5) | <0.001 | |  |
| **Score system** |  |  | |  |  | |  |
| SOFA | 3 (2-5) | 3 (2-4) | | 5 (3-9) | <0.001 | |  |
| **Interventions, n (%)** |  |  | |  |  | |  |
| Ventilation | 8189 (75.6) | 7142 (78.5) | | 1047 (60.6) | <0.001 | |  |
| RRT | 1114 (10.3) | 671 (7.4) | | 443 (25.7) | <0.001 | |  |
| Vasopressor | 4738 (43.8) | 3726 (40.9) | | 1012 (58.6) | <0.001 | |  |
| **Comorbidities, n (%)** |  |  | |  |  | |  |
| COPD | 2665 (24.6) | 2640 (29.0) | | 25 (1.4) | <0.001 | |  |
| Hypertension | 6833 (63.1) | 6017 (66.1) | | 816 (47.2) | <0.001 | |  |
| Liver disease | 1782 (16.5) | 1656 (18.2) | | 126 (7.3) | <0.001 | |  |
| Renal failure | 2797 (25.8) | 2574 (28.3) | | 223 (12.9) | <0.001 | |  |
| Cerebrovascular disease | 1651 (15.3) | 1383 (15.2) | | 268 (15.5) | <0.001 | |  |
| Diabetes | 3424 (31.6) | 2997 (32.9) | | 427 (24.7) | <0.001 | |  |
| Cancer | 1575 (14.5) | 1282 (14.1) | | 293 (17.0) | <0.001 | |  |
| **Primary outcome** |  |  | |  |  | |  |
| 28-day mortality, n (%) | 2286 (21.1) | 2010 (22.1) | | 276 (16.0) | <0.001 | |  |
| **Secondary outcome** |  |  | |  |  | |  |
| 90-day mortality, n (%) | 2952 (27.3) | 2658 (29.2) | | 294 (17.0) | <0.001 | |  |
| ICU mortality, n (%) | 1306 (12.1) | 1124 (12.4) | | 182 (10.5) | 0.034 | |  |
| In-hospital mortality, n (%) | 1915 (17.7) | 1612 (17.7) | | 303 (17.5) | 0.864 | |  |
| ICU length of stay, day | 3.1 (1.8-6.1) | 3.0 (1.8-5.8) | | 4.0 (1.0-9.0) | 0.001 | |  |
| Hospital length of stay, day | 9.0 (5.3-16.4) | 8.4 (5.1-14.7) | | 15.0 (8.0-27.0) | <0.001 | |  |

Abbreviation: SBP- systolic blood pressure; DBP- diastolic blood pressure; MAP- mean arterial pressure; Cr- creatinine; WBC- white blood cell count; PT-prothrombin time; INR- international normalized ratio; SOFA- Sequential Organ Failure Assessment; RRT- Renal Replacement Therapy; COPD- Chronic Obstructive Pulmonary Disease.

**Table S2: Association of serum magnesium and 28-day mortality by multivariable-adjusted logistic analysis in the American MIMIC-IV cohort.**

| Categories | Unadjusted | | Model 1 | | Model 2 | |
| --- | --- | --- | --- | --- | --- | --- |
|  | OR (95%CI) | P value | OR (95%CI) | P value | OR (95%CI) | P value |
| Serum total magnesium | 1.97 (1.53-2.55) | <0.001 | 2.03 (1.57-2.64) | <0.001 | 1.31 (1.02-1.68) | 0.036 |
| Serum total magnesium quintiles | |  |  |  |  |  |
| Q1 | 0.87 (0.72-1.04) | 0.124 | 0.86 (0.71-1.03) | 0.102 | 0.87 (0.72-1.05) | 0.144 |
| Q2 | Ref |  | Ref |  | Ref |  |
| Q3 | 1.06 (0.92-1.23) | 0.411 | 1.07 (0.92-1.23) | 0.391 | 1.02 (0.88-1.18) | 0.780 |
| Q4 | 1.28 (1.11-1.49) | 0.001 | 1.28 (1.10-1.49) | 0.001 | 1.15 (0.98-1.34) | 0.082 |
| Q5 | 1.54 (1.32-1.80) | <0.001 | 1.51 (1.29-1.78) | <0.001 | 1.18 (1.00-1.40) | 0.047 |
| P for trend | <0.001 | | <0.001 | | 0.002 | |

| Categories | Model3 | | | | | Model 4 | | |  |
| --- | --- | --- | --- | --- | --- | --- | --- | --- | --- |
|  | OR (95%CI) | | P value | | | OR (95%CI) | | P value |  |
| Serum total magnesium | 1.37 (1.07-1.77) | | 0.014 | | | 1.47 (1.12-1.93) | | 0.005 |  |
| Serum total magnesium quintiles | |  | |  | | |  | | |
| Q1 | 0.86 (0.71-1.04) | | 0.113 | | | 0.86 (0.71-1.04) | | 0.121 |  |
| Q2 | Ref | |  | | | Ref | |  |  |
| Q3 | 1.02 (0.88-1.18) | | 0.834 | | | 1.07 (0.91-1.24) | | 0.418 |  |
| Q4 | 1.13 (0.97-1.31) | | 0.129 | | | 1.19 (1.01-1.39) | | 0.033 |  |
| Q5 | 1.20 (1.01-1.41) | | 0.033 | | | 1.27 (1.07-1.50) | | 0.007 |  |
| P for trend | 0.001 | | | | <0.001 | | | |  |

OR, odds ratio; CI, confidence interval; Ref, reference.

Model 1 was adjusted for age, gender, ethnicity.

Model 2 was adjusted for the confounders included in model 1 plus SOFA.

Model 3 was adjusted for the confounders included in model 1 plus laboratory tests (white blood cell count, hemoglobin, platelets, serum creatinine, international normalized ratio, serum potassium).

Model 4 was adjusted for the confounders included in model 3 plus comorbidities (hypertension, liver disease, renal failure, diabetes, malignant cancer, chronic pulmonary disease and cerebrovascular disease).

**Table S3: Association of serum magnesium and 28-day mortality by multivariable-adjusted logistic analysis in the Chinese institutional cohort.**

| Categories | Model3 | | | Model 4 | |
| --- | --- | --- | --- | --- | --- |
|  | OR (95%CI) | P value | OR (95%CI) | | P value |
| Serum total magnesium | 1.75 (0.96-3.20) | 0.067 | 1.93 (1.04-3.57) | | 0.037 |
| Serum total magnesium quintiles | |  |  | |  |
| G1 | 1.02 (0.66-1.58) | 0.932 | 1.52 (0.96-2.42) | | 0.074 |
| G2 | Ref |  | Ref | |  |
| G3 | 0.98 (0.55-1.73) | 0.935 | 1.12 (0.63-2.01) | | 0.694 |
| G4 | 1.33 (0.85-2.08) | 0.214 | 1.38 (0.87-2.18) | | 0.166 |
| G5 | 1.45 (0.92-2.28) | 0.111 | 1.52 (0.96-2.42) | | 0.074 |
| P for trend | 0.047 | | | 0.028 | |

| Categories | Unadjusted | | | Model 1 | | Model 2 | |
| --- | --- | --- | --- | --- | --- | --- | --- |
|  | OR (95%CI) | P value | OR (95%CI) | | P value | OR (95%CI) | P value |
| Serum total magnesium | 2.00 (1.16-3.46) | 0.013 | 2.03 (1.15-3.59) | | 0.014 | 1.11 (0.59-2.09) | 0.739 |
| Serum total magnesium quintiles | |  |  | |  |  |  |
| G1 | 1.04 (0.68-1.58) | 0.861 | 1.03 (0.67-1.58) | | 0.892 | 1.13 (0.72-1.78) | 0.603 |
| G2 | Ref |  | Ref | |  | Ref |  |
| G3 | 0.95 (0.55-1.65) | 0.857 | 0.95 (0.54-1.67) | | 0.863 | 1.01 (0.56-1.82) | 0.968 |
| G4 | 1.46 (0.96-2.24) | 0.079 | 1.44 (0.93-2.22) | | 0.100 | 1.24 (0.78-1.97) | 0.365 |
| G5 | 1.62 (1.06-2.47) | 0.025 | 1.59 (1.04-2.45) | | 0.034 | 1.25 (0.78-1.99) | 0.353 |
| P for trend | 0.005 | | | 0.008 | | 0.303 | |

OR, odds ratio; CI, confidence interval; Ref, reference.

Model 1 was adjusted for age, gender, BMI.

Model 2 was adjusted for the confounders included in model 1 plus SOFA.

Model 3 was adjusted for the confounders included in model 1 plus laboratory tests (white blood cell count, hemoglobin, platelets, serum creatinine, international normalized ratio, serum potassium, total bilirubin).

Model 4 was adjusted for the confounders included in model 3 plus comorbidities (hypertension, liver disease, renal failure, diabetes, malignant cancer, chronic pulmonary disease and cerebrovascular disease).

**Figure S1:** **Univariate analysis of risk factors associated with 28-day mortality in patients with sepsis**


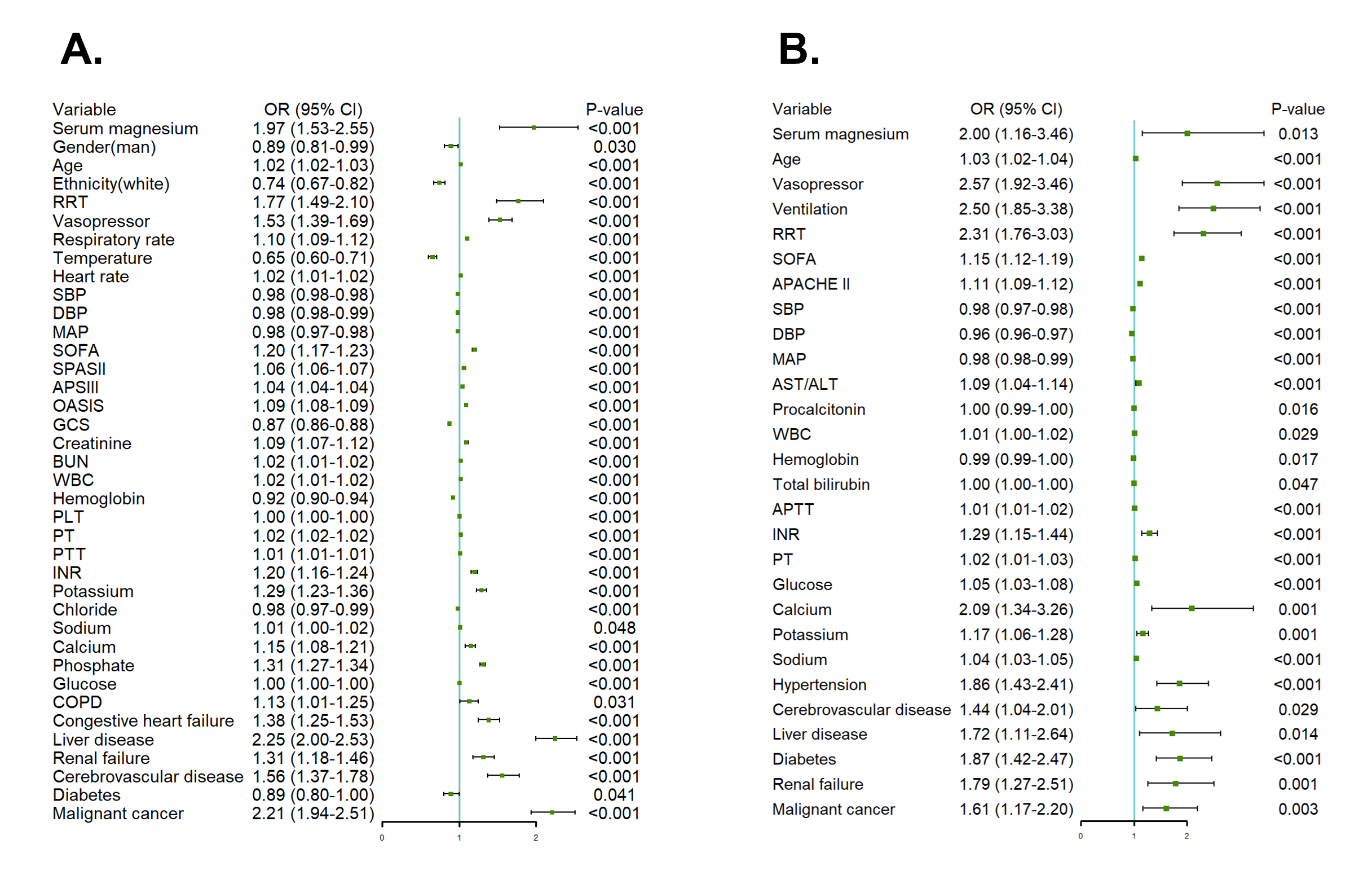


**A． MIMIC-IV Cohort B． Institutional Cohort**

Abbreviation: SBP- systolic blood pressure; DBP- diastolic blood pressure; MAP- mean arterial pressure; Cr- creatinine; BUN- blood urea nitrogen; WBC- white blood cell count; PT-prothrombin time; PTT- partial thromboplastin time; APTT- partial thromboplastin time; INR- international normalized ratio; SAPSII-Simplified Acute Physiology Score II; APSIII- Acute Physiology and Chronic Health Evaluation III; SOFA- Sequential Organ Failure Assessment; APACHE II - Acute Physiology and Chronic Health Evaluation II;

RRT- Renal Replacement Therapy; COPD- Chronic Obstructive Pulmonary Disease.

**Figure S2:** **Receiver operating characteristic curve of serum magnesium for sepsis**


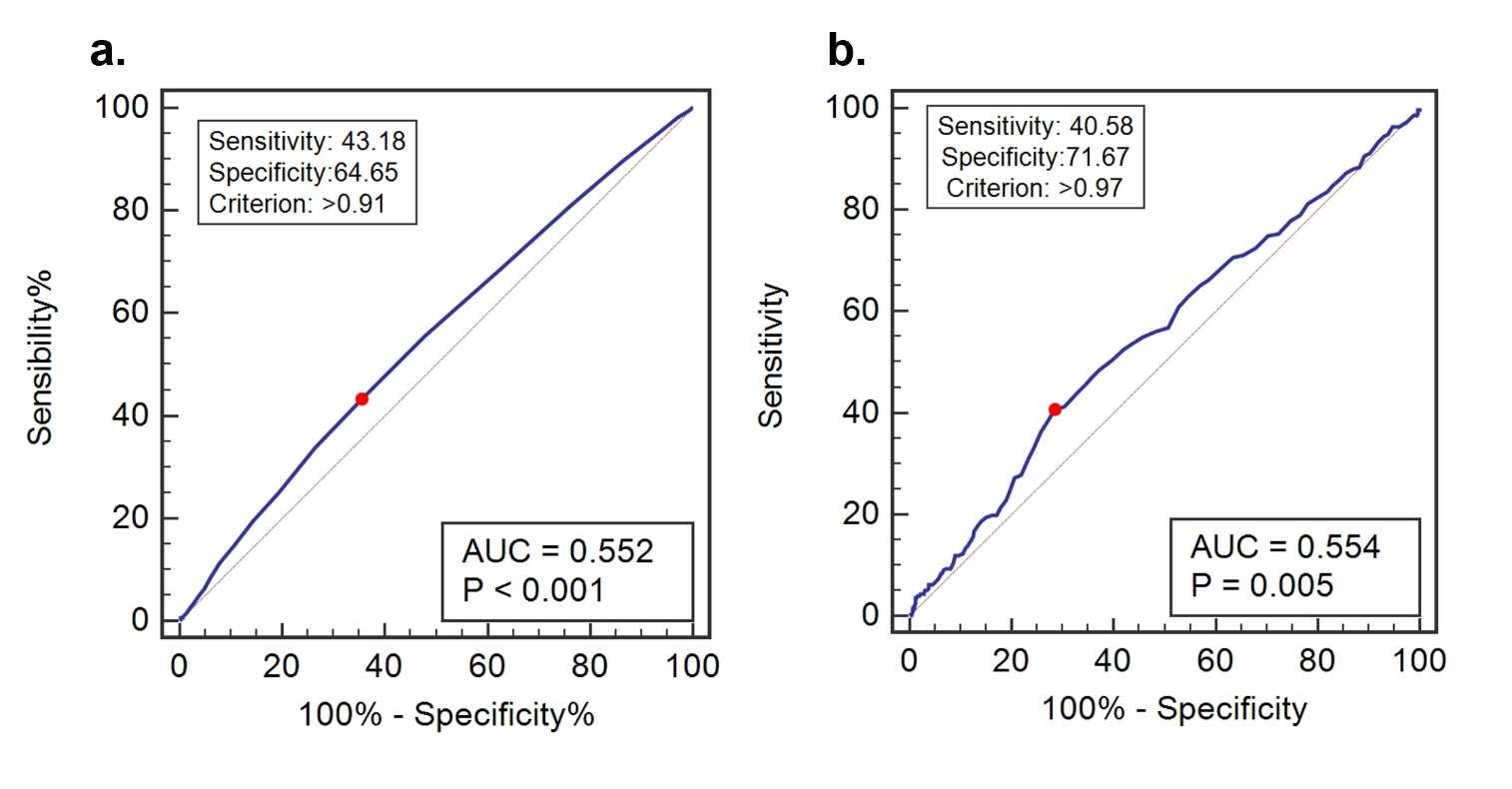


**A． MIMIC-IV Cohort B． Institutional Cohort**

AUC, area under the curve.

**Figure S3: Diagnostic criteria for sepsis**

**
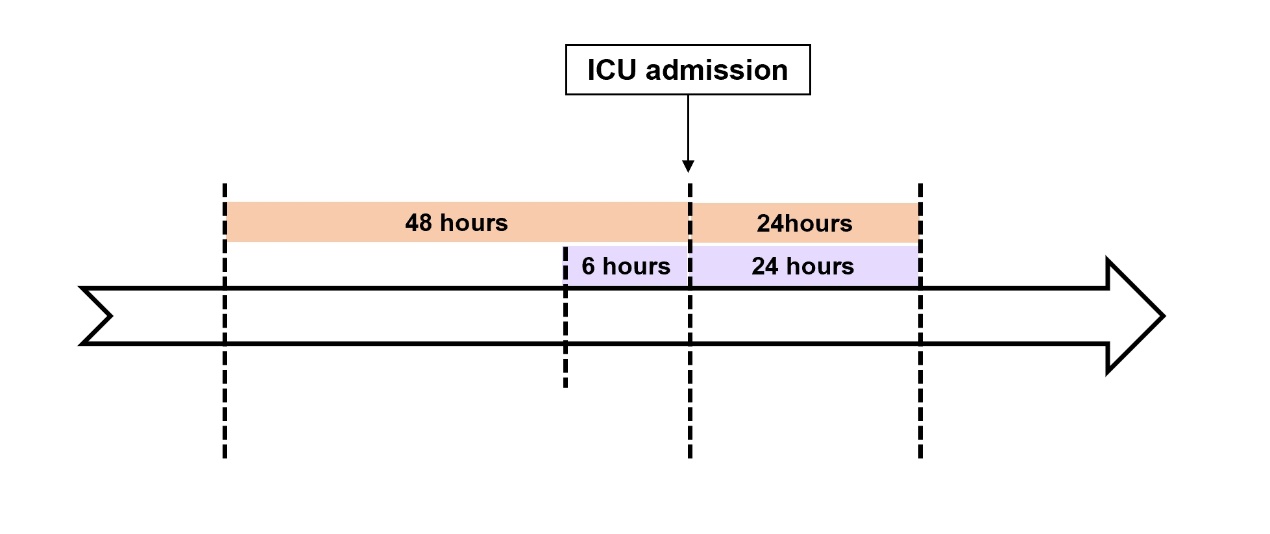
**

Patients with suspected or documented infection combined SOFA score no less than 2 points were diagnosed sepsis (Diagnosis time between 6 hours before ICU admission and 24 hours after ICU admission).

Patients who used magnesium sulfate within the first 48 hours of ICU admission and the following 24 hours were excluded.

The maximum serum magnesium level between 6 hours before ICU admission and 24 hours after ICU admission was recorded.

Abbreviations: SOFA, Sequential Organ Failure Assessment; ICU, intensive care unit.

**Figure S4: Percentage of missing data of variable**


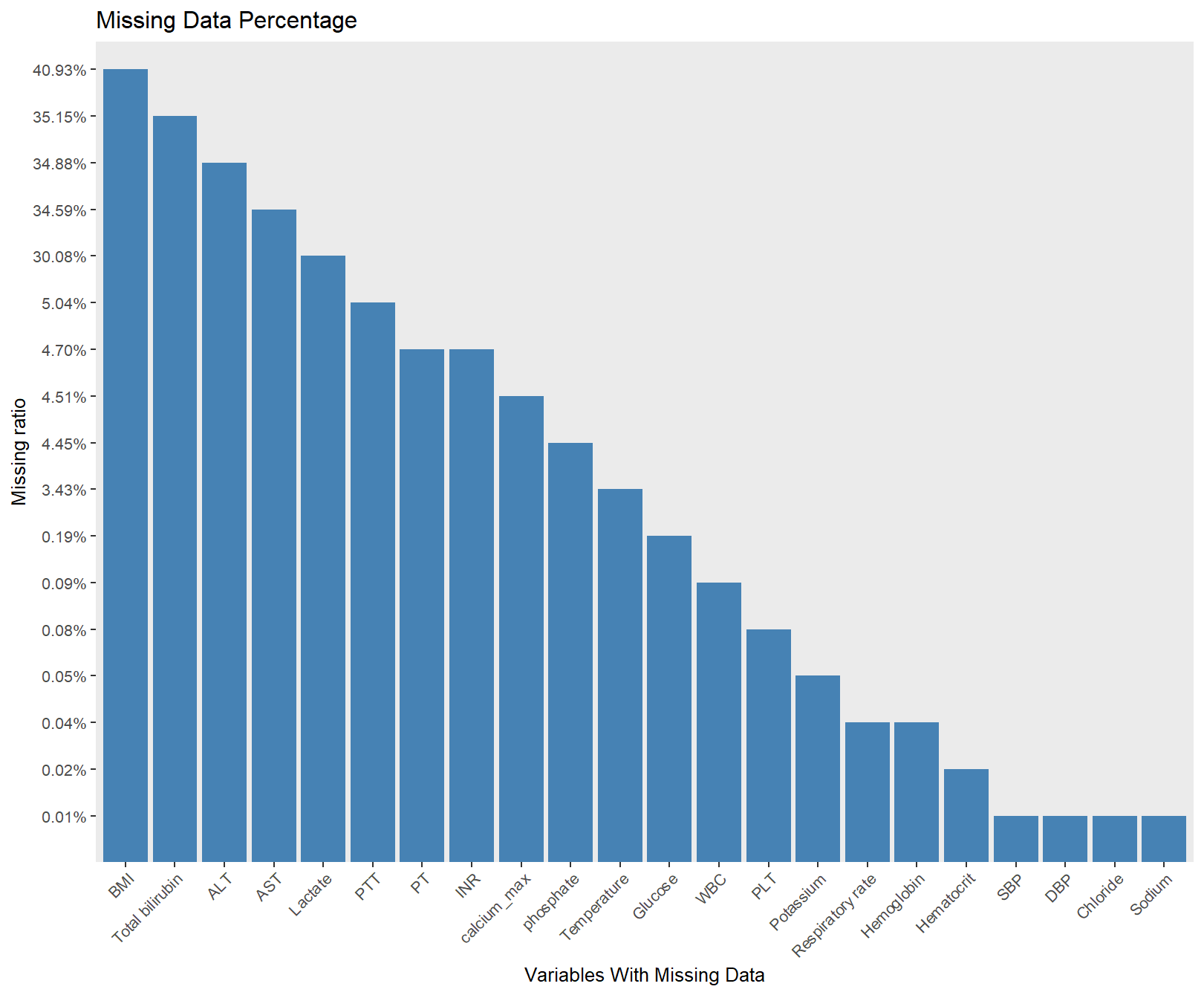


We eliminated variables with more than 20% missing values, including BMI, total bilirubin, ALT, AST, and lactate. The remaining variables containing missing values were interpolated using the MICE package random forest method of R software.
